# Accurate Identification of Reentrant Circuit and Critical Isthmus of an Atrial Tachycardia Over the Posterior Wall of the Left Atrium Requiring a 1.4-second Single Radiofrequency Energy Application

**DOI:** 10.19102/icrm.2021.120104S

**Published:** 2021-01-15

**Authors:** Hakop Hrachian, Juliana Rios

**Affiliations:** ^1^Mount Sinai Medical Center, Miami Beach, FL, USA; ^2^Abbott, Chicago, IL, USA

**Keywords:** Atrial tachycardia, high-density mapping, radiofrequency ablation, repeat ablation

A 61-year-old man with longstanding persistent atrial fibrillation (AF) since 2010 presented to the clinic, having undergone cardioversion for AF in 2016 and AF ablation (pulmonary vein isolation, posterior left atrium isolation, and cavotricuspid isthmus ablation) in July 2017. He developed atrial tachycardia (AT) a few months after the AF ablation procedure and underwent AT ablation (LA posterior wall) in October 2017. However, he experienced early recurrence, requiring a second cardioversion (for AT) and treatment with dofetilide in 2018. He remained primarily in sinus rhythm until September 2020, when he developed persistent AT again, which recurred after a third cardioversion attempt in early October 2020.

In late October 2020, the patient underwent repeat AT ablation using the Advisor™ HD Grid Mapping Catheter, Sensor Enabled™, which confirmed macro-reentrant AT originating from the posterior wall of the left atrium **(Video 1)**. A very narrow isthmus was identified precisely; delivery of one radiofrequency energy application with 35 W of power terminated the tachycardia in 1.4 seconds.

However, postablation mapping showed scattered areas of live tissue over the posterior wall communicating with the left atrium. Mapping with the Advisor™ HD Grid catheter was able to identify the reentrant circuit accurately and further ablation was performed in sinus rhythm to achieve complete isolation of the posterior wall. In addition, all remaining potentials were eliminated, having been missed using other catheters during the previous two ablation sessions. Isolation persisted after 30 minutes of observation and the infusion of isoproterenol during the postablation period.

## Figures and Tables

**Figure 1:**
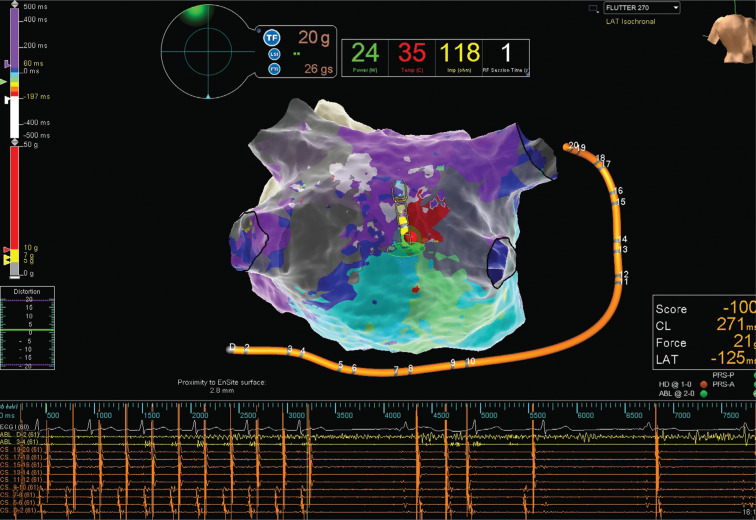
Left atrial posterior wall.

**Video 1.** Propagation animation of the local activation mapping performed with the Advisor™ HD Grid catheter showing the macro-reentrant AT (cycle length: 270 ms) found on the posterior wall of the left atrium. The critical isthmus with very slow conduction can be seen on the right lower part of the posterior wall. The low voltage identifier was set at 0.1 mV.

